# Analysis of dead-core formation in catalytic reaction and diffusion processes with generalized diffusion flux

**DOI:** 10.1038/s41598-022-26786-8

**Published:** 2022-12-23

**Authors:** Piotr Skrzypacz, Bek Kabduali, Alua Kadyrbek, Sławomir Szafert, Vsevolod Andreev, Boris Golman

**Affiliations:** 1 Department of Mathematics, School of Sciences and Humanities, Nazarbayev University, 010000 Astana, Kazakhstan; 2 Department of Chemistry, University of Wrocław, 14 F. Joliot-Curie, 50-383 Wrocław, Poland; 3 Department of Heat Power Setups, Faculty of Energy and Electrical Engineering, Chuvash State University, Cheboksary, Russia 428015; 4 Department of Chemical and Materials Engineering, School of Engineering and Digital Sciences, Nazarbayev University, 010000 Astana, Kazakhstan

**Keywords:** Chemical engineering, Heterogeneous catalysis, Applied mathematics

## Abstract

Dead-core and non-dead-core solutions to the nonlinear diffusion–reaction equation based on the generalized diffusion flux with gradient-dependent diffusivity and the power-law reaction kinetics in catalyst slabs are established. The formation of dead zones where the reactant concentration vanishes is characterized by the critical Thiele modulus that is derived as a function of reaction order and diffusion exponent in the generalized diffusion flux. The effects of reaction order and diffusion exponent on the reactant concentration distribution in the slab and dead-zone length are analyzed. It is particularly demonstrated that by contrast to the model based on the standard Fick’s diffusion, dead-core solutions exist in the case of first-order reactions. Also, the relationship between critical Thiele moduli for models based on the generalized and standard Fick’s diffusion fluxes is established.

## Introduction

Nonlinear diffusion-reaction equations are important mathematical tools to understand and quantify phenomena arising in heterogeneous catalysis^1–11^. In petrochemical and chemical industries, chemical reactions are commonly carried out in reactors containing catalyst pellets^12,13^. The character of solutions to diffusion-reaction problems is largely influenced by various factors including process parameters, type and rate of reaction, type of diffusion, the morphology of catalyst pellets, and reactor design. In particular cases, reactions may cease in some parts of a catalyst pellet due to the lack of reactant. This phenomenon is caused by the insufficient supply of reactant to the pellet interior by diffusion. Temkin^14^ defined such zones as “dead cores” or “dead zones”. Dead zones have been observed in different fields of chemical engineering, including propylene hydrogenation on a commercial catalyst, power production in a microbial fuel cell, and bioreaction in catalytic particles with immobilized enzymes, see references in^8^. Most authors of quite numerous engineering papers about dead zone phenomena in heterogeneous catalysis consider models based on the Fickian diffusion flux^15^

$$\begin{aligned} j = -D\nabla c, \end{aligned}$$

where the diffusivity *D* is assumed to be constant or spatially dependent. Concerning generalizations of Fickian diffusion, we first note that Fick’s law is usually an approximation, and a natural next step to obtain a more accurate analysis is to consider some nonlinear generalization. However, works characterizing dead zone formation in models with non-Fickian diffusion are very rare. Recently^9^, the dead zone formation in catalyst slabs with power-law kinetics and external mass transfer was investigated for the non-standard model based on the diffusion flux of the form

$$\begin{aligned} j = -D(c)\nabla c=-D_{\textrm{eff}}\nabla (c^m), \end{aligned}$$

where the diffusivity *D* is a power-law function of concentration $D(c)=mD_{\textrm{eff}}\,c^{m-1}$, $m>0$, and $D_{\textrm{eff}}$ is the constant. The diffusion-reaction model based on the defined above concentration-dependent diffusivity can possess dead-core solutions for reactions with power-law kinetics even if the reaction exponent is not of fractional order^9^.

In this work, the Fickian diffusion is generalized as follows

$$\begin{aligned} {j=-D(\nabla c)\nabla c=-D_{\textrm{eff}}|\nabla c|^{p-2}\nabla c} \end{aligned}$$

where the diffusivity $D(\nabla c)=D_{\textrm{eff}}|\nabla c|^{p-2}$, $p>1$, is a power-law function of the concentration gradient. The number *p* will be called the diffusion exponent in the following. The gradient-dependent diffusivity of above type was introduced by Philip^16^, and it is sometimes called the Philip *n*-diffusion^17^ due to the original setting $j=-D_{\textrm{eff}}|\nabla c|^{n-1}\nabla c$, $n>0$. Notice that this type of diffusion leads to *p*-Laplacian problems whose analysis attracts since 50ies remarkable attention from mathematicians^2,3,18–20^. Despite the numerous papers on the *p*-Laplacian equation and its recent applications in areas such as non-Newtonian fluids, turbulent flows in porous media, glaciology, game theory or image analysis, there are only a few engineering-oriented papers concerning the validity of the *p*-Laplacian model and its applications for solving transport problems of chemical species driven by the gradient-dependent nonlinear diffusion^21–25^.

The concentration gradient dependency of surface diffusivity during the adsorption of water in microporous silica gels was reported by Kruckels et al.^25^. Recently, Partopour et al.^26^ confirmed that the concentration gradient dependency of diffusivity can influence reaction and, as a result, the reactant and product concentration profiles in the pellets, especially for pellets with small pores, reduced porosity, and high tortuosity. The industrially important catalytic reactions with deactivation due to coke deposition are one example of such systems.

In the present paper, we will consider the catalytic chemical reactions following power law kinetics with fractional order. The methanol synthesis from carbon dioxide and hydrogen on supported copper-zinc oxide catalysts^27^, syngas production from dry methane reforming on nickel catalyst^28^, hydrogen production by the catalytic decomposition of hydrous hydrazine^29^, and conversion of ethanol into 1,3-butadiene on hemimorphite-HfO2/SiO2 catalyst^30^ are just a few examples of such reactions.

In order to characterize the formation of dead zones, the concept of the critical Thiele modulus was introduced in^6,31^. The Thiele modulus describes the relationship between diffusion and reaction rates in porous catalyst pellets. In the case when the Thiele modulus exceeds its critical value, the dead core will exist^31^. Previously, many researchers attempted to derive approximate and exact dead-core and non-dead-core solutions for the reaction-diffusion problems with the standard Fick’s diffusion. For example, Aris^6^ derived the dead-core and non-dead-core solutions to the pellets of planar geometry without external mass transfer. Andreev^31^ stated necessary and sufficient conditions for the occurrence of dead zones and derived the critical Thiele modulus for cylindrical and spherical pellets with external mass transfer. The semi-analytic dead-core and non-dead-core solutions to diffusion-reaction equations for pellets of planar, cylindrical, and spherical geometries and with external mass transfer were proposed in^8^. The case of the dead-zone formation for slightly non-isothermal reactions was studied in^32^. Notice that in the abovementioned models the diffusivity dependence on the temperature was neglected. The dead-zone formation in models with temperature-dependent diffusivity was investigated in^7^.

The main objective of the present paper is to propose a practical method for characterizing dead-core and non-dead-core solutions to diffusion-reaction problems based on the generalized gradient-dependent diffusion flux of the power-law type. The paper is organized as follows. In “Mathematical model” section, the mathematical model for the nonlinear diffusion-reaction equation is presented. The critical Thiele modulus and dead-core solutions are established in “Critical Thiele modulus and dead-core solutions” section, and the non-dead-core solutions are derived in terms of Gauss hypergeometric functions in “Non-dead-core solutions” section. The comparison of critical Thiele moduli for generalized and standard diffusion models is presented in “Comparison of critical Thiele moduli” section. In “Illustration of results” section, the obtained results are illustrated and discussed. Finally, the outcomes of the paper are concluded in “Conclusion” section.

## Mathematical model

Let us consider a single reaction

$$\begin{aligned} \text {Reactant}\,A\rightarrow \text {Products} \end{aligned}$$

in a catalyst slab of half-thickness *R*, and let $r_{p}\in [0,R]$ be the distance from the pellet center. We assume that the generalized diffusion flux is given by

1 $$\begin{aligned} j_A = -D_{A}\,\left| \frac{dC_{A}}{dr_{p}}\right| ^{p-2}\frac{dC_{A}}{dr_{p}},\quad p>1, \end{aligned}$$

where $C_{A}(r_{p})$ is the concentration of the reactant *A* in the catalyst slab, $D_{A}$ denotes the effective diffusion coefficient of reactant *A*, and $p>1$ is called the diffusion exponent. If $p=2$, the generalized diffusion flux given by (1) becomes the standard Fick’s law^15^.

The steady-state reaction-diffusion equation in the catalyst slab reads as follows

2 $$\begin{aligned} D_{A}\frac{d}{dr_{p}}\left( \left| \frac{dC_{A}}{dr_{p}}\right| ^{p-2}\frac{dC_{A}}{dr_{p}}\right) = r(C_{A}),\quad r_p\in (0,R), \end{aligned}$$

where $r(C_{A})$ corresponds to the power-law reaction kinetics such that

3 $$\begin{aligned} r(C_{A}) = kC_{A}^{n},\quad n\geqslant 0. \end{aligned}$$

Here, the reaction rate is assumed to follow the power-law kinetics with $n\geqslant 0$ being the reaction order, and $k>0$ the reaction rate constant. Equation (2) is complemented by the boundary condititions

4 $$\begin{aligned} \frac{dC_{A}}{dr_{p}} \Big |_{r_{p}=0}=0\quad \text {and}\quad C_{A}(R) = C_{A,b}, \end{aligned}$$

where $C_{A,b}$ is the concentration of reactant *A* in the bulk phase.

Integrating both sides of Eq. (2) yields

5 $$\begin{aligned} D_{A}\left| \frac{dC_{A}}{dr_{p}}(r_p)\right| ^{p-2}\frac{dC_{A}}{dr_{p}}(r_p) = k \int _{0}^{r_{p}} C_{A}^{n}(s) \,ds, \end{aligned}$$

where $k \int _{0}^{r_{p}} C_{A}^{n}(s) \,ds \geqslant 0$ due to Eq. (3) for $C_{A}\geqslant 0$. Obviously, $\left| \frac{dC_{A}}{dr_{p}}\right| ^{p-2} \geqslant 0$, and consequently $\frac{dC_{A}}{dr_{p}} \geqslant 0$. Therefore, Eq. (2) can be rewritten as

6 $$\begin{aligned} D_{A}\frac{d}{dr_{p}}\left[ \left( \frac{dC_{A}}{dr_{p}}\right) ^{p-1} \right] = kC^{n}_{A}. \end{aligned}$$

Let us introduce the dimensionless distance $x=r_p/R$, dimensionless concentration $u=C_A/C_{A,b}$, and the Thiele modulus

7 $$\begin{aligned} \phi _{p,n} = \sqrt{\frac{R^pkC^{n-p+1}_{A,b}}{D_{A}}}. \end{aligned}$$

Then, Eq. (6) together with the boundary conditions by Eq. (4) are transformed into the dimensionless form

8 $$\begin{aligned} \frac{d}{dx}\left( \left( \frac{du}{dx} \right) ^{p-1} \right)&= \phi ^2_{p,n}u^{n}\quad \text {in}\quad (0,1),\\ \frac{du}{dx}(0)&=0,\quad u(1)=1. \end{aligned} $$

The Thiele modulus defined by Eq. (7) will be applied in this study to analyze the formation of dead zones. Notice that for the case of $p=2$, the Thiele modulus by Eq. (7) is given as

$$\begin{aligned} \phi _{2,n} = \sqrt{\frac{R^{2}kC_{A,b}^{n-1}}{D_{A}}}, \end{aligned}$$

which coincides with the definition of the Thiele modulus for problems based on the standard Fick’s law^6,8^. If the Thiele modulus $\phi _{2,n}$ exceeds a certain threshold, a dead zone of length $x_{dz}$ can be formed close to the pellet center. Its length depends on the particle size and shape, effective diffusivity, mass transfer coefficient, bulk reactant concentration, reaction order, and reaction rate constant. On the dead-zone boundary, the following conditions are satisfied:

9 $$\begin{aligned} u(x_{dz}) = 0\quad \text {and}\quad \frac{du}{dx}(x_{dz}) = 0. \end{aligned}$$

Furthermore, in the case when the diffusion flux obeys the standard Fick’s law, the necessary and sufficient conditions for the existence of dead zones are given as

10 $$\begin{aligned} n \in (-1,1) \end{aligned}$$

and

11 $$\begin{aligned} \phi _{2,n} \ge \phi _{2,n}^{*} = \sqrt{\frac{2}{1-n} \left( \frac{1+n}{1-n} \right) }, \end{aligned}$$

respectively^8,31^. Here, $\phi _{2,n}^*$ denotes the critical Thiele modulus corresponding to the initiation of dead zone. The dead core solution to the diffusion–reaction problem for the critical Thiele modulus for the diffusion flux given by the standard Fick’s law was derived as^8,31^

12 $$\begin{aligned} u(x) = x^{\frac{2}{1-n}}. \end{aligned}$$

## Critical Thiele modulus and dead-core solutions

In the following, we will derive a dead-core solution to the diffusion-reaction problem with generalized diffusion flux by Eq. (8). Multiplying both sides of Eq. (8) by $\frac{du}{dx}$ results in

13 $$\begin{aligned} (p-1)\left( \frac{du}{dx}\right) ^{p-1}\frac{d^2u}{dx^2} = \phi ^{2}_{p,n}u^{n}\frac{du}{dx}. \end{aligned}$$

Then, integrating both sides of Eq. (13) implies that

14 $$\begin{aligned} \frac{p-1}{p}\left( \frac{du}{dx} \right) ^{p} = \frac{\phi ^{2}_{p,n}}{n+1}u^{n+1}+K, \end{aligned}$$

where the integration constant *K* is given by

15 $$\begin{aligned} K=-\frac{\phi ^{2}_{p,n}}{n+1}u^{n+1}(0) \end{aligned}$$

due to the boundary condition at $x=0$. In the critical case of $\phi _{p,n}=\phi _{p,n}^*$ when the formation of dead zone starts, $u(0) = 0$ and consequently $K=0$. Therefore,

16 $$\begin{aligned} \frac{p-1}{p}\left( \frac{du}{dx} \right) ^{p} = \frac{(\phi ^*_{p,n})^2}{n+1}u^{n+1}. \end{aligned}$$

Integrating the separable differential equation (16) yields

17 $$\begin{aligned} \int \limits _{0}^{u(x)} \frac{ds}{s^{\frac{n+1}{p}}} =\int \limits _{0}^{x}\left( \frac{p}{(p-1)(n+1)} \right) ^{\frac{1}{p}}(\phi ^*_{p,n})^{\frac{2}{p}} ds, \end{aligned}$$

where $$\frac{n+1}{p} < 1$$, i.e., $$p>n+1$$. Consequently,

18 $$\begin{aligned} \frac{p}{p-n-1}u^{\frac{p-n-1}{p}}(x) =\left( \frac{p}{(p-1)(n+1)} \right) ^{\frac{1}{p}}(\phi ^*_{p,n})^{\frac{2}{p}}x, \end{aligned}$$

from which we infer

19 $$\begin{aligned} u(x)=\left( \frac{p-n-1}{p}\right) ^{\frac{p}{p-n-1}}\left( \frac{p}{(p-1)(n+1)} \right) ^{\frac{1}{p-n-1}}(\phi ^*_{p,n})^{\frac{2}{p-n-1}}x^{\frac{p}{p-n-1}}. \end{aligned}$$

Thus, the critical Thiele modulus for reaction-diffusion problems with generalized diffusion is derived as

20 $$\begin{aligned} \phi ^{*}_{p,n} = \left( \frac{p}{p-n-1} \right) ^{\frac{p}{2}}\left( \frac{(p-1)(n+1)}{p} \right) ^{\frac{1}{2}} \end{aligned}$$

due to the boundary condition at $x=1$. If $p=2$, then the critical Thiele modulus is given by

21 $$\begin{aligned} \phi ^{*}_{2,n} = \frac{2}{1-n}\frac{\sqrt{n+1}}{\sqrt{2}} = \frac{\sqrt{2(n+1)}}{1-n}, \end{aligned}$$

which coincides with Eq. (11), cf.^6,8^.

From Eqs. (19) and (20) it follows that the separatrix is of the form

22 $$\begin{aligned} u(x) = x^{\frac{p}{p-n-1}} \end{aligned}$$

for $0 \leqslant n < p-1$. Notice that there exists no dead-core solution if $p-1\leqslant n$. If $p=2$, then the separatrix is given by $u(x) = x^{\frac{2}{1-n}}$ which coincides with Eq. (12), cf.^8^.

If $\phi _{p,n}>\phi _{p,n}^*$, then the dead-core solution reads as follows

23 $$\begin{aligned} u(x) = {\left\{ \begin{array}{ll} \left( \frac{p-n-1}{p}\right) ^{\frac{p}{p-n-1}} \left( \frac{p}{(p-1)(n+1)}\right) ^{\frac{1}{p-n-1}}\phi _{p,n}^{\frac{2}{p-n-1}} (x-x_{dz})^{\frac{p}{p-n-1}}, &{} \quad x_{dz}\leqslant x \leqslant 1,\\ 0, &{} \quad 0\leqslant x\leqslant x_{dz}. \end{array}\right. } \end{aligned}$$

Employing the boundary condition at $x=1$, we can derive the length of the dead-zone as

24 $$\begin{aligned} x_{dz} = 1 - \frac{p}{p-n-1}\left( \frac{(p-1)(n+1)}{p}\right) ^{\frac{1}{p}}\phi _{p,n}^{-\frac{2}{p}}. \end{aligned}$$

If $p=2$, then Eq. (24) is transformed to

25 $$\begin{aligned} x_{dz}= 1 -\sqrt{\frac{2(1+n)}{\phi _{2,n}^{2}(1-n)^{2}}}, \end{aligned}$$

which coincides with the result obtained for the model based on the standard Fick’s diffusion, see^8^.

## Non-dead-core solutions

In the following, we will consider the case of $p-1< n$ when the two-point boundary value problem for Eq. (8) possesses solutions without dead zones. Let

$$\begin{aligned} u_0=u(0)\ne 0. \end{aligned}$$

In the case of non-dead-core solution, the integration constant *K* in Eq. (14) is given by

26 $$\begin{aligned} K = -\frac{\phi _{p,n}^{2}}{n+1}u^{n+1}_{0}\ne 0. \end{aligned}$$

Therefore, it follows from Eq. (14) that

27 $$\begin{aligned} \frac{du}{dx} = \left( \frac{p}{p-1} \left( \frac{\phi _{p,n}^{2}}{n+1}u^{n+1}_{0}\right) \right) ^{\frac{1}{p}}\left( \left( \frac{u}{u_{0}}\right) ^{n+1}-1\right) ^{\frac{1}{p}}. \end{aligned}$$

Integrating both sides of above equation results in

28 $$\begin{aligned} \int \limits _{u_{0}}^{u(x)} \left[ \left( \frac{s}{u_{0}}\right) ^{n+1}-1\right] ^{-\frac{1}{p}} \,ds = \left( \frac{p}{p-1}\frac{\phi _{p,n}^{2}}{n+1}u^{n+1}_0\right) ^{\frac{1}{p}}x. \end{aligned}$$

Substituting $\xi = \frac{s}{u_{0}}$ in the integral in Eq. (28) yields

29 $$\begin{aligned} \int \limits _{1}^{\frac{u(x)}{u_{0}}} [\xi ^{n+1}-1]^{-\frac{1}{p}}\,d\xi = \left( \frac{p}{p-1} \frac{\phi _{p,n}^{2}}{n+1}u^{n-p+1}_{0}\right) ^{\frac{1}{p}}x. \end{aligned}$$

Let $z=1-\xi ^{-n-1}$, $\xi = (1-z)^{-\frac{1}{n+1}}$, $d\xi =\frac{1}{n+1}(1-z)^{-\frac{1}{n+1}-1}$. Then, Eq. (29) can be rewritten as

30 $$\begin{aligned}  \int \limits _{0}^{1-\left( \frac{u_{0}}{u(x)}\right) ^{n+1}} z^{\frac{p-1}{p}-1}(1-z)^{\frac{1}{p}-\frac{1}{n+1}-1}\,dz&= \left( \frac{p}{p-1}\frac{\phi _{p,n}^{2}}{n+1}u^{n-p+1}_{0}\right) ^{\frac{1}{p}}(n+1)\, x, \end{aligned} $$

from which it follows that for $x=1$ the unknown concentration $u_0$ at the pellet center can be obtained from the following eqution

31 $$\begin{aligned} \int \limits _{0}^{1-u_{0}^{n+1}} z^{\frac{p-1}{p}-1}(1-z)^{\frac{1}{p}-\frac{1}{n+1}-1}\,dz = \left( \frac{p}{p-1}\frac{\phi _{p,n}^{2}}{n+1}u^{n-p+1}_{0}\right) ^{\frac{1}{p}}(n+1) . \end{aligned}$$

The integral on the left side of Eq. (31) can be expressed in terms of the incomplete Beta function

$$\begin{aligned}B(a,b,z) = \int _{0}^{z} t^{a-1} (1-t)^{b-1}dt\end{aligned}$$

and the Gauss hypergeometric function

$$\begin{aligned} F(a,b;c;z) = \sum _{j=0}^{\infty } \frac{(a)_{j}(b)_{j}}{(c)_{j}}\frac{z^{j}}{j!}, \end{aligned}$$

where $|z| < 1$ and $(q)_{j}$ denotes the Pochhammer symbol defined by

$$\begin{aligned} (q)_{j} = {\left\{ \begin{array}{ll} 1 &{} \text {for}\quad j=0,\\ q(q+1)...(q+j-1)>1 &{} \text {for}\quad j>1, \end{array}\right. } \end{aligned}$$

cf.^33^. In the case when $\frac{1}{p}-\frac{1}{n+1}>0$, i.e., $p-1<n$, Eq. (31) can be rewritten as

32 $$\begin{aligned}&\left( \frac{p}{(p-1)(n+1)} \left( 1 - u_{0}^{n+1}\right) \right) ^{\frac{p-1}{p}}\cdot F\left( \frac{p-1}{p},1-\frac{1}{p}+\frac{1}{n+1};\frac{p-1}{p}+1;1-u_0^{n+1}\right) \\&= \phi _{p,n}^{\frac{2}{p}}u^{\frac{n-p+1}{p}}_{0} \end{aligned}$$

by using the relation

$$\begin{aligned} B(a,b,z) = \frac{z^{a}}{a}F(a, 1-b; a+1; z). \end{aligned}$$

If $p=2$, then $u_0$ satisfies

33 $$\begin{aligned} \left( \frac{2}{n+1} \left( 1 - u_{0}^{n+1}\right) \right) ^{\frac{1}{2}}F\left( \frac{1}{2},\frac{1}{2}+\frac{1}{n+1};\frac{3}{2};1-u_{0}^{n+1}\right) = \phi _{2,n}\, u_0^{\frac{n-1}{2}}, \end{aligned}$$

which coincides with the result from^8^ for the model based on the standard Fick’s diffusion.

Once the unknown concentration $u_0$ at the pellet center is iteratively determined from the algebraic nonlinear Eq. (32), the concentration *u*(*x*) is given implicitly by the following formula

34 $$\begin{aligned}&F\left( \frac{p-1}{p},1-\frac{1}{p}+\frac{1}{n+1};\frac{p-1}{p}+1;1 - \left( \frac{u_{0}}{u(x)}\right) ^{n+1}\right) \\&= \phi _{p,n}^{\frac{2}{p}}u^{\frac{n-p+1}{p}}_{0}\left( \frac{p}{(p-1)(n+1)} \left( 1 - \left( \frac{u_{0}}{u(x)}\right) ^{n+1}\right) \right) ^{\frac{1-p}{p}}\, x, \end{aligned}$$

which coincides for $p=2$ with the result obtained in^8^.

## Comparison of critical Thiele moduli

**Figure 1:**
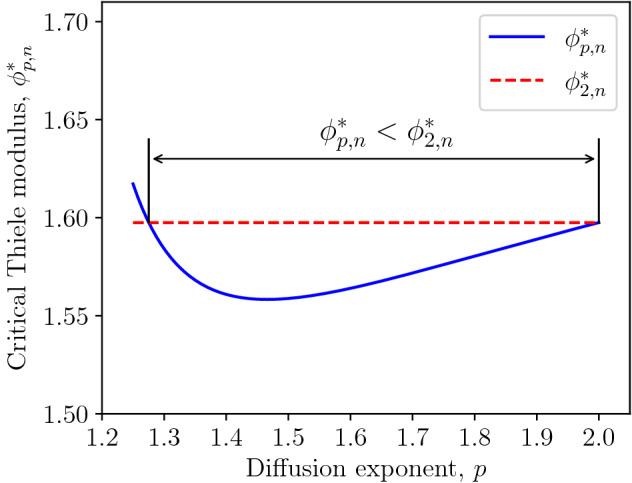
Thiele modulus $\phi ^*_{p,n}$ (solid line) and the threshold $\phi _{2,n}^*$ (dashed line) for $n=0.08$.

In this section, we will compare the critical Thiele modulus for the model based on the generalized diffusion with the corresponding threshold for the model based on the standard Fick’s diffusion. For $1<p<2$, the generalized diffusion flux is much stronger than the diffusion flux based on the Fick’s law. Therefore, the reaction rate must be higher to ensure the formation of dead zone. One could intuitively expect that the critical Thiele modulus $\phi _{p,n}^*$ by Eq. (20) for the generalized model exceeds $\phi _{2,n}^*$ by Eq. (21) for the standard Fick’s model. In the following, we will demonstrate that this is not the case, i.e., there is some range of $p\in (1,2)$ such that $\phi _{p,n}^*<\phi _{2,n}^*$ if the reaction exponent *n* is below a certain threshold.

The logarithmic derivative of the critical Thiele modulus from Eq. (20) with respect to the exponent *p* is given by

35 $$\begin{aligned} \frac{\partial \phi _{p,n}^*}{\partial p} = \left\{ \frac{1}{2}\ln {\left( \frac{p}{p-n-1}\right) } +\frac{p}{2(p-1)}-\frac{p}{2(p-n-1)}-\frac{1}{2p}\right\} \phi _{p,n}^*. \end{aligned}$$

We observe that $\frac{\partial \phi _{p,n}^*}{\partial p}(2,n)\geqslant 0$ if $n\leqslant n^*$, where $n^*$ satisfies the equation

$$\begin{aligned} \ln {\left( \frac{2}{1-n^*}\right) }-\frac{2}{1-n^*}+\frac{3}{2}=0, \end{aligned}$$

which is equivalent to

36 $$\begin{aligned} -\frac{2}{1-n^*}e^{-\frac{2}{ 1-n^{*}}}=-e^{-\frac{3}{2}}. \end{aligned}$$

The solution of Eq. (36) is given by

37 $$\begin{aligned} n^*=1+\frac{2}{W_{-1}(-e^{-\frac{3}{2}})}\approx 0.15170, \end{aligned}$$

where $W_{-1}:[-1/e,0)\rightarrow (-\infty ,-1]$ denotes the second branch of the Lambert W function^34,35^ which is defined as a solution $y(x)\leqslant -1$ to the equation $ye^{y}=x$ for $x\in [-1/e,0)$.

We conclude that

$$\begin{aligned} \phi _{p,n}^*\geqslant \phi _{2,n}^*\quad \text {for}\quad 1\geqslant p-1 >n\geqslant n^*. \end{aligned}$$

Otherwise, there is a range of $p\in (1,2)$ such that $\phi _{p,n}^*<\phi _{2,n}^*$, as illustrated in Fig. 1. The critical Thiele modulus $\phi ^*_{p,n}$ for the model based on the generalized diffusion is less than the critical Thiele modulus $\phi ^*_{2,n}$ for the model based on the standard Fick’s diffusion for the range of the diffusion exponent *p* from approximately 1.27 to 2 if the reaction exponent $n=0.08$.

A remarkable relationship between the critical Thiele moduli for the models based on the generalized and Fick diffusions can be established by transforming the *p*-Laplacian problem by Eq. (8) into the problem based on the standard Fick’ diffusion. From (13) and (16) it follows that the separatrix *u*(*x*) satisfies

38 $$\begin{aligned}  \frac{d^2u}{dx^2}&= \left[ \left( \phi ^*_{p,n}\right) ^{\frac{2}{p}}\left( \frac{p}{n+1}\right) ^{\frac{2-p}{2p}}(p-1)^{-\frac{1}{p}}\right] ^2u^{n+\frac{(2-p)(n+1)}{p}}\quad \text {in}\quad (0,1),\\ \frac{du}{dx}(0)&=0,\quad u(1)=1, \end{aligned}$$

which constitutes the diffusion-reaction model based on the standard Fick’s diffusion and reaction exponent $n+\frac{(2-p)(n+1)}{p}$. The critical Thiele modulus for the standard Fick’s diffusion model by Eq. (38) is given by

39 $$\begin{aligned} \phi _{2,n+\frac{(2-p)(n+1)}{p}}^*=\frac{\sqrt{p(n+1)}}{p-1-n} \end{aligned}$$

due to Eq. (21). Consequently, the critical Thiele moduli $\phi _{p,n}^*$ and $\phi _{2,n+\frac{(2-p)(n+1)}{p}}^*$ satisfy the relation

40 $$\begin{aligned} \phi _{p,n}^*=\left( \phi _{2,n+\frac{(2-p)(n+1)}{p}}^*\right) ^{\frac{p}{2}}\left( \frac{p}{n+1}\right) ^{\frac{p-2}{4}}\sqrt{p-1}, \end{aligned}$$

which can be verified by substituting $\phi _{2,n+\frac{(2-p)(n+1)}{p}}^*$ from Eq. (39) into Eq. (40). Consequently,

41 $$\begin{aligned} \phi _{p,n}^*&=\left( \frac{\sqrt{p(n+1)}}{p-1-n}\right) ^{\frac{p}{2}}\left( \frac{p}{n+1}\right) ^{\frac{p}{4}-\frac{1}{2}}(p-1)^{\frac{1}{2}}=\left( \frac{p}{p-1-n}\right) ^{\frac{p}{2}}\left( \frac{(p-1)(n+1)}{p}\right) ^{\frac{1}{2}}. \end{aligned}$$

Eq. (41) coincides with Eq. (20) already derived in “Critical Thiele modulus and dead-core solutions” section, confirming the relation by Eq. (40) between the critical Thiele moduli for models based on the generalized and standard diffusion Fick’s fluxes.

## Illustration of results

**Figure 2:**
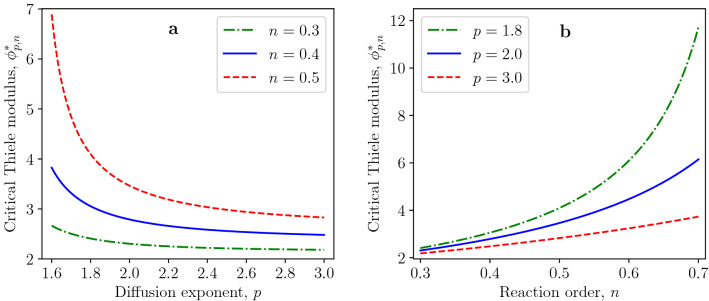
Dependence of critical Thiele modulus $\phi ^*_{p,n}$ on (**a**) diffusion exponent *p* and (**b**) reaction order *n*.

In the following, we will demonstrate the effects of the diffusion exponent *p* and reaction order *n* on the critical Thiele modulus $\phi ^*_{p,n}$ by Eq. (20). The critical Thiele modulus decreases with increasing values of the diffusion exponent, as shown in Fig. 2a. This tendency is more pronounced for higher values of reaction order. The increase of critical Thiele modulus for decreasing values of the diffusion exponent can be explained by the fact that the dimensionless generalized diffusion flux at $x=1$ is given by

$$\begin{aligned} j\arrowvert _{x=1}=\left( \frac{du}{dx}\right) ^{p-1}\biggl \arrowvert _{x=1}=\left( \frac{p}{p-n-1}\right) ^{p-1}=\left( \frac{1}{1-\frac{n+1}{p}}\right) ^{p-1} \end{aligned}$$

for *u*(*x*) being the separatrix defined in Eq. (22). The generalized flux at $x=1$ increases for decreasing values of $p>1$ which follows from

$$\begin{aligned} \frac{\partial j\arrowvert _{x=1}}{\partial p}=-\left( \frac{1}{1-\frac{n+1}{p}}\right) ^{p-1}\ln {\left( \frac{1}{1-\frac{n+1}{p}}\right) }\cdot \frac{n+1}{p-n-1}<0 \end{aligned}$$

for $p>n+1$. The effect of the reaction order *n* on the critical Thiele modulus $\phi _{p,n}^*$ is opposite to the effect of diffusion exponent, as presented in Fig. 2b. The growing fractional reaction order *n* leads to the decreasing rate of reactant consumption by the reaction. Thus, the dead zone is formed at higher values of the Thiele modulus.

**Figure 3:**
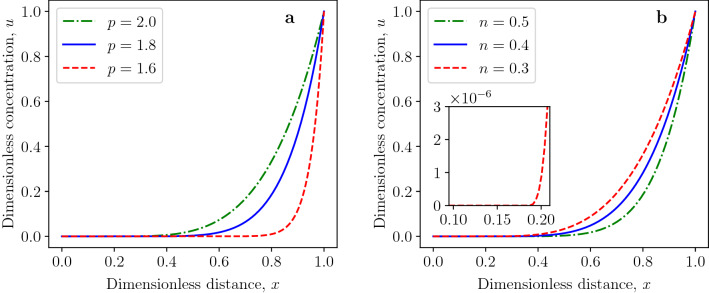
Profiles of dead-core solutions for $\phi _{p,n}=1.2\,\phi _{p,n}^*$ and for (**a**) various diffusion exponents and fixed reaction order $n=0.5$; (**b**) various reaction orders and fixed diffusion exponent $p=1.8$.

**Figure 4:**
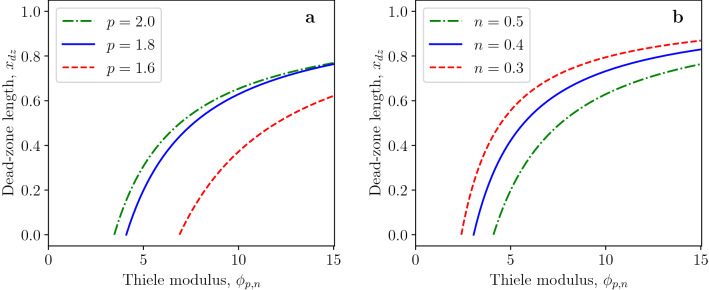
Effect of Thiele modulus on dead-zone length for (**a**) various diffusion exponents and (**b**) reaction orders.

The effects of the diffusion exponent and reaction order on the concentration profiles of dead-core solutions are illustrated in Fig. 3a, b, respectively. The dead-zone length significantly increases with decreasing diffusion exponent for the Thiele modulus $\phi _{p,n}=1.2\,\phi _{p,n}^*$, as shown in Fig. 3a. This can be deduced from Eqs. (20) and (24). Namely, the dead-zone length for $\phi _{p,n}=1.2\,\phi _{p,n}^*$ is given by

42 $$\begin{aligned} x_{dz}=1-(1.2)^{-\frac{2}{p}}, \end{aligned}$$

which is a monotonically decreasing function of the reaction exponent $p>1$. The dead-zone length for the varying reaction exponent is constant $x_{dz}=0.1834$ for the Thiele modulus $\phi _{p,n}=1.2\,\phi _{p,n}^*$ and $p=1.8$ due to Eq. (42), as shown in Fig. 3b. For the same *n* and different *p*, the dimensionless concentration is smallest for the smallest *p*, whereas, for the same *p* and different reaction order *n*, the concentration is highest for the smallest value of the reaction order *n*. The effects of Thiele modulus $\phi _{p,n}$ on the dead-zone length for various diffusion and reaction exponents are presented in Fig. 4a, b, respectively. In both cases, the dead-zone length increases with increasing Thiele modulus.

**Figure 5:**
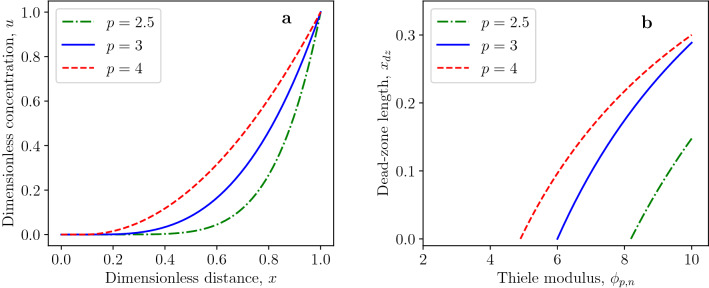
(**a**) Profiles of dead-core solutions for the first-order reaction ($n=1$) and $\phi _{p,n}= 1.2\,\phi _{p,n}^*$. (**b**) Effect of Thiele modulus on dead-zone length for first-order reaction ($n=1$).

**Figure 6:**
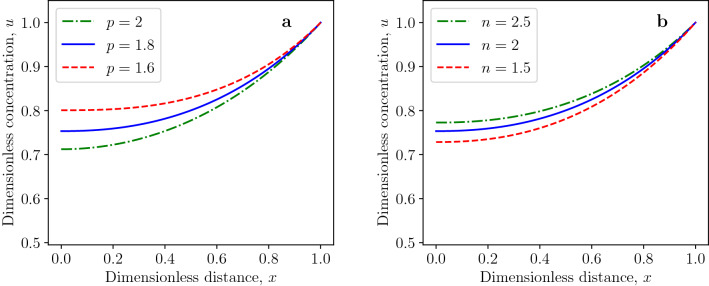
Profiles of non-dead-core solutions for (**a**) $n=2$, $\phi _{p,n}=1$ and (**b**) $p=1.8$, $\phi _{p,n}=1$.

Another interesting result obtained is the fact that the dead zone can exist even for first-order reactions ($n=1$) when $p>2$. The necessary condition for the existence of dead zone when the diffusion flux obeys the standard Fick’s law is $n \in (-1, 1)$. This necessary condition does not hold in the case of the *p*-Laplacian equation since the dead zone can exist for $n=1$, as demonstrated in Fig. 5. The effects of diffusion exponent and reaction order on profiles of non-dead-core solutions are shown in Fig. 6. Figures 6a and 4a confirm that the generalized diffusion flux increases with decreasing diffusion exponent *p* which leads to larger concentration values.

## Conclusion

Dead-core and non-dead-core solutions to the reaction and diffusion processes with generalized diffusion flux and with power-law kinetics in catalyst slabs were derived. The investigated *p*-Laplacian model is the generalization of the model based on the standard Fick’s diffusion considering diffusivity’s dependence on the concentration gradient. It was found that for the constant reaction order $n\geqslant 0$ as the diffusion exponent $p>1$ increases, the critical Thiele modulus $\phi ^{*}_{p,n}$ decreases. However, in the case of the constant diffusion exponent *p* as the reaction order *n* increases, the critical Thiele modulus $\phi ^{*}_{p,n}$ increases as well. Furthermore, the reactant concentration distribution in the slab is affected by the reaction order *n* and diffusion exponent *p*. The studied case of $\phi _{p,n}=1.2\phi _{p,n}^*$ shows that for the fixed reaction order *n* and varying diffusion exponent *p*, the dimensionless concentration is smallest for the smallest values of *p*, whereas, for the fixed diffusion exponent *p* and varying reaction exponent *n*, the concentration is highest for the smallest reaction order *n*. The dead-zone length becomes larger for decreasing *n* and increasing *p*. Finally, in the case of generalized diffusion, the dead-core solution to the *p*-Laplacian diffusion-reaction equation exists for first-order reactions ($n=1$), while in the case of the standard Fick’s diffusion, the dead-core solution does not exist if $n=1$.

## Data Availability

The datasets generated during and/or analyzed during the current study are available from the corresponding author upon reasonable request.
